# Poor humoral and T-cell response to two-dose SARS-CoV-2 messenger RNA vaccine BNT162b2 in cardiothoracic transplant recipients

**DOI:** 10.1007/s00392-021-01880-5

**Published:** 2021-07-09

**Authors:** René Schramm, Angelika Costard-Jäckle, Rasmus Rivinius, Bastian Fischer, Benjamin Müller, Udo Boeken, Assad Haneya, Zdenek Provaznik, Cornelius Knabbe, Jan Gummert

**Affiliations:** 1 Klinik für Thorax- und Kardiovaskularchirurgie, Herz und Diabeteszentrum NRW, Universitätsklinik, Ruhr-Universität Bochum, Georgstr. 11, 32545 Bad Oeynhausen, Germany; 2 Klinik für Kardiologie, Angiologie Und Pneumologie, Universitätsklinikum Heidelberg, Im Neuenheimer Feld 672, 69120 Heidelberg, Germany; 3 Institut für Transfusions- Und Labormedizin, Herz Und Diabeteszentrum NRW, Universitätsklinik, Ruhr-Universität Bochum, Georgstr. 11, 32545 Bad Oeynhausen, Germany; 4 Klinik für Herzchirurgie, Universitätsklinikum Düsseldorf, Heinrich Heine Universität Düsseldorf, Moorenstr. 5, 40225 Düsseldorf, Germany; 5 Klinik für Herznahe- und Gefäßchirurgie, Universitätsklinikum Schleswig-Holstein, Arnold-Heller-Str. 3, 24105 Kiel, Germany; 6 Klinik für Herz-, Thorax- Und Herznahe Gefäßchirurgie, Universitätsklinikum Regensburg, Franz-Josef-Strauß-Allee 11, 93053 Regensburg, Germany

**Keywords:** Covid-19 infection, BioNTech/Pfizer (BNT162b2) vaccine, Immunocompromised patients, Transplant recipients, Immunogenicity

## Abstract

**Aims:**

Immunocompromised patients have been excluded from studies of SARS-CoV-2 messenger RNA vaccines. The immune response to vaccines against other infectious agents has been shown to be blunted in such patients. We aimed to analyse the humoral and cellular response to prime-boost vaccination with the BNT162b2 vaccine (Pfizer-BioNTech) in cardiothoracic transplant recipients.

**Methods and results:**

A total of 50 transplant patients [1–3 years post heart (42), lung (7), or heart–lung (1) transplant, mean age 55 ± 10 years] and a control group of 50 healthy staff members were included. Blood samples were analysed 21 days after the prime and the boosting dose, respectively, to quantify anti-SARS-CoV-2 spike protein (S) immunoglobulin titres (tested by Abbott, Euroimmun and RocheElecsys Immunoassays, each) and the functional inhibitory capacity of neutralizing antibodies (Genscript). To test for a specific T-cell response, heparinized whole blood was stimulated with SARS-CoV-2 specific peptides, covering domains of the viral spike, nucleocapsid and membrane protein, and the interferon-γ release was measured (QuantiFERON Monitor ELISA, Qiagen). The vast majority of transplant patients (90%) showed neither a detectable humoral nor a T-cell response three weeks after the completed two-dose BNT162b2 vaccination; these results are in sharp contrast to the robust immunogenicity seen in the control group: 98% exhibited seroconversion after the prime dose already, with a further significant increase of IgG titres after the booster dose (average > tenfold increase), a more than 90% inhibition capability of neutralizing antibodies as well as evidence of a T-cell responsiveness.

**Conclusions:**

The findings of poor immune responses to a two-dose BNT162b2 vaccination in cardiothoracic transplant patients have a significant impact for organ transplant recipients specifically and possibly for immunocompromised patients in general. It urges for a review of future vaccine strategies in these patients.

## Introduction

The Covid-19 pandemic caused by severe acute respiratory syndrome coronavirus 2 (SARS-CoV-2) has a widespread impact on health, including a substantial mortality among older adults and patients with pre-existing health conditions [1]. Solid organ transplant recipients are considered a group at increased risk: although not associated with a higher infection rate, maybe due to high adherence to self-care measures preliminary data suggest an increased risk of severe disease and death in case of infection [2–5].

Vaccination has emerged as a key tool for controlling the pandemic health crisis by preventing severe disease and mortality and by increasing population immunity.

Four vaccines have been approved by the European Medicines Agency (EMA) on base of the phase 3 clinical efficacy studies showing good safety and immunogenicity [6–9] However, immunocompromised patients have been excluded from these studies.

In spite of lacking data about the novel concept of mRNA vaccines in organ transplant recipients, national and international transplant societies have recommended earliest possible vaccination for all recipients > 3–6 months post-transplant (unless recently treated with lymphocyte-depleting agents) and national vaccination strategies have suggested prioritized treatment for this potentially vulnerable group [10–12].

The immune response to other types of vaccines have been shown to be blunted in immunosuppressed patients [13, 14].

To gain more insights in the immunogenicity of mRNA vaccines under immunosuppressive therapy, we analysed the antibody as well as the T-cell response after the first and second dose of the BNT162b2 vaccination in cardiothoracic organ transplant recipients.

## Methods

### Study participants and data collection

Transplant recipients (Tx) who had been offered vaccination with the BNT162b2 vaccine (Pfizer-BioNTech) were recruited through their German transplant centres to participate in this prospective cohort and those who received an offer for SARS-CoV-2 vaccination (independently of the study, according to the German priority guideline) were included. The study was approved by the local Ethical committee of the Heart and Diabetes Centre Nordrhein-Westfalen (HDZ) in Bad Oeynhausen, Germany (Reg.-No 2021-742), and participants provided written informed consent.

Healthy members of the medical staff of the HDZ who were offered the vaccination with BNT162b2 in-hospital served as controls. Samples were collected in accordance with the German Act on Medical Devices for the collection of human residual material. All staff members gave written informed consent. The study was registered in the German Clinical Trials Register (DRKS00024199).

Blood samples were captured: pre-vaccination (Tx group), 21 days after the first vaccine dose and 21 days after the second vaccine dose (Tx and control group), respectively.

### Serologic testing

#### Determination of anti-SARS-CoV-2 IgG antibodies (Abbott)

The commercial SARS-CoV-2 IgG II Quant assay (Abbott, Lake Forrest, IL, USA) is a chemiluminescent microparticle immunoassay (CMIA) which was used for the quantitative measurement of IgG antibodies against the spike receptor-binding domain (RBD) of SARS-CoV-2 in human serum on the Alinity I system. Data were expressed in WHO standardized units BAU (binding antibody unit) per ml. According to the manufacturer’s recommendation, values below 7.1 BAU/ml were regarded as negative whereas values equal to or above 7.1 BAU/ml were interpreted as positive for IgG antibodies against SARS-CoV-2.

#### Determination of anti-SARS-CoV-2 IgG antibodies (Euroimmun)

Two commercial ELISAs (Euroimmun, Lübeck, Germany) were used to test for antibodies to the S1 domain of the SARS-CoV-2 spike protein (IgG). For quantitative determination of IgG, data were expressed in relative Units per ml (RU/ml). Values below 10 RU/ml were regarded as negative whereas values above 10 RU/ml were interpreted as positive as stated by the manufacturer.

#### Determination of anti-SARS-CoV-2 IgG antibodies (RocheElecsys)

The Elecsys Anti-SARS-CoV-2 S assay (Roche, Penzberg, Germany) is a commercially available immunoassay using a recombinant RBD of the S-Antigen representing protein for the quantitative determination of high-affinity antibodies to SARS-CoV-2 on a Roche cobas e411 platform. For quantitative determination of IgG, data were expressed in Units per ml (U/ml). Values smaller than 0.8 U/ml were interpreted as negative for anti-SARS-CoV-2 antibodies and positive otherwise following the manufacturers’ instructions.

#### Determination of neutralizing antibodies against SARS-CoV-2

The presence of neutralizing antibodies against SARS-CoV-2 was determined using the cPass™ SARS-CoV-2 Neutralization Antibody Detection KIT (GenScript, Piscataway Township, USA) and performed according to the manufacturer’s instructions. The inhibition capability was calculated as follows:

$$ {\text{Inhibition}} = \left( {1 - \frac{{\text{OD value of sample}}}{{\text{OD value of negative control}}}} \right) \times 100\% $$

According to the manufacturer, values greater than or equal to 20% were considered positive concerning neutralizing antibodies.

### Tests for T-cell response

#### Stimulation of immune cells using SARS-CoV-2 peptides

To test for a cellular immune response, immune cells from heparinized whole blood were stimulated with SARS-CoV-2 specific peptides (Miltenyi Biotec, Bergisch-Gladbach, Germany), covering domains of the viral spike, nucleocapsid, and membrane protein (final concentration of each peptide: 1 µg/ml). Treatment of whole blood with water served as negative controls.

#### Determination of interferon-γ in plasma

Interferon-γ (IFN-γ) release was evaluated using a commercial ELISA (QuantiFERON Monitor ELISA, Quiagen, Hilden, Germany), modified as previously described to allow for rapid and reliable analysis with a standard microplate-reader not requiring manual plate-coating [15, 16]. IFN-γ values of unstimulated controls were subtracted from the stimulated samples.

### Statistical analysis

Results are presented as mean ± standard deviation for continuous variables with normal distribution, median [interquartile range (IWR), 25th to 75th percentiles] for continuous variables without normal distribution, and number (percentage) for categorical data. Student’s *t* test was used to compare normally distributed continuous variables between two groups. The Mann–Whitney *U* test was used to analyse non-normally distributed data. Statistical analyses were performed in Python using the SciPy package. Figures were created in Python using the seaborn and matplotlib libraries. Statistical tests are two-sided, and *p* values < 0.05 were considered to be statistically significant.

## Results

### Patient characteristics

Fifty transplant recipients (Tx) and 50 healthy staff members serving as control group were included in the study. The Tx group had a higher percentage of male patients than the control group (64% vs. 34%, *p* < 0.0001) and a higher average age (55 ± 10 vs 47 ± 10 years, *p* < 0.0001).

The Tx group was homogenic with respect to the time since transplant, all having been transplanted between 1 and 3 years before study inclusion [median 689 (501; 859) days].

Most Tx patients (92%) were on an immunosuppressive regimen with a calcineurin inhibitor, combined with mycofenolate acid or mofetil (Table 1).

**Table 1:** Demographic and clinical characteristics of transplant recipients

	Transplant recipients (*n*)
	(*n* = 50)
Age group (years)	
18–39	5
40–59	32
> 60	13
Sex	
Male	32
Female	18
Type of organ transplantation	
Heart	42
Lung	7
Heart–lung	1
Time since transplantation (months)	
10–12	1
12–24	25
24–36	24
Type of maintenance immunosuppression	
Tacrolimus/MMF	41
Cyclosporin/MMF	5
Tacrolimus/mTOR-inhibitor	4

*MMF* mycofenolate mofetil, *mTOR *mammalian target of rapamycin

### Previous SARS-CoV-2 infection

None of the Tx patients had detectable Anti-SARS-CoV-2 IgG-titres (Abbot) prior to the first vaccination dose and none had been tested positive for Anti-SARS-CoV-2 before. All individuals of the control group of healthy staff members had undergone weekly pooled PCR analyses of nasopharyngeal swaps and none had tested positive prior to the first vaccination (nor during the 6 weeks following the prime-boost vaccination).

### B-cell response following prime-boost SARS-CoV-2 vaccination

#### Anti-SARS-CoV-2 IgG titres

**Fig. 1:**
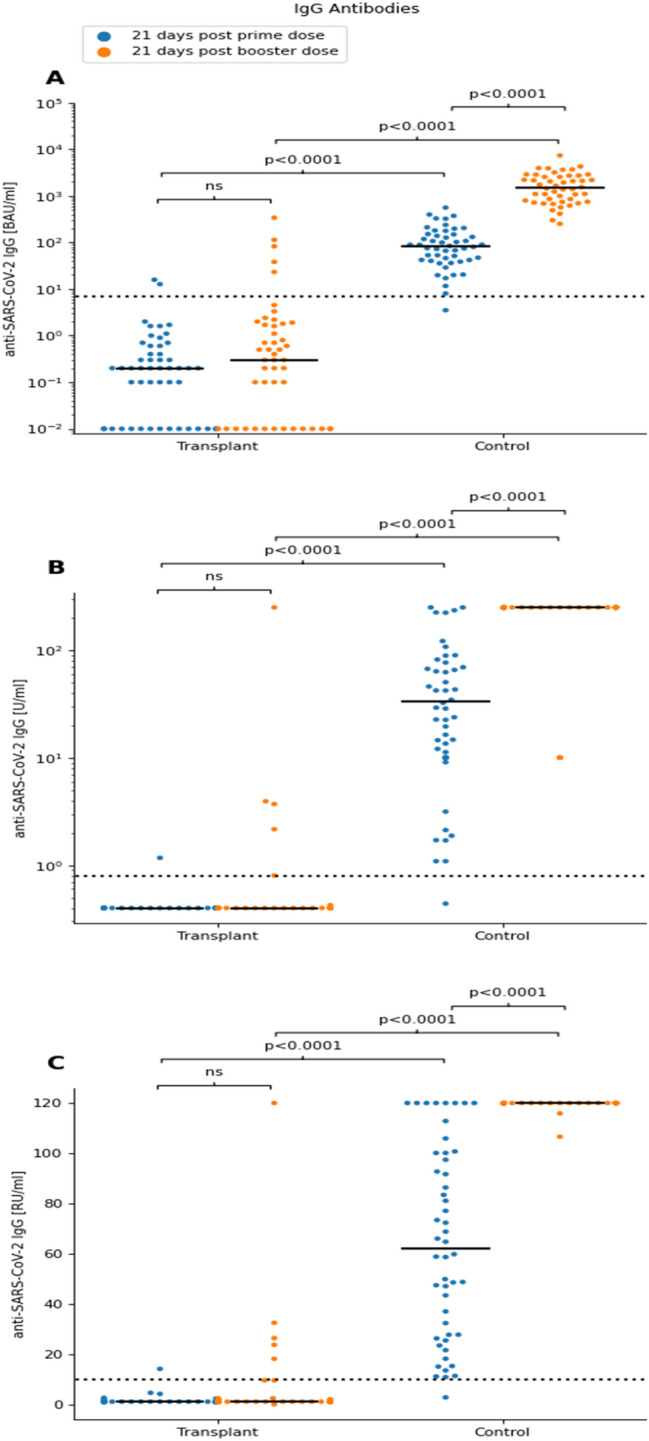
Serological responses to BNT162b2 vaccination in transplant recipients and healthy controls (n = 50 each): IgG-Antibodies. Samples drawn 21 days after the prime (blue dots) and the booster dose (orange dots). Scatter plots show: **A** Anti-SARS-CoV-2 IgG, given in binding antibody units per ml ([BAU/ml]; Abbott). **B** Anti-SARS-CoV-2 IgG, given in Units per ml ([U/ml]; RocheElecsys). **C** Anti-SARS-CoV-2 IgG, given in Relative Units per ml [(RU/ml); Euroimmun]. Statistical analysis was by Mann–Whitney Test. Horizontal solid lines show Medians. Horizontal dotted lines show cut-off values according to manufacturers. *ns *non-significant, *IgG *immunoglobulin G

Anti-SARS-CoV-2 IgG titres above the cut-off value of 7.1 BAU/ml (Abbot-ELISA) were detected in all but one control subjects one 21 days after the prime dose [Median 82 (41;149) BAU/ml]. 21 days after the booster dose the titres had markedly increased in all individuals [median 1417 (732; 2589) BAU/ml, *p* < 0.0001 vs prime dose]. Notably, there was a broad range of Anti-SARS-CoV-2 IgG titres within the control group at both timepoints (range 8–559 and 251–7351 BAU/ml after the prime and boost dose, respectively; Fig. 1a). Analyses with the immunoassays of Roche and Euroimmun revealed comparable results (Fig. 1b, c): titres above the cut-off values were detectable in all but one individuals after the prime dose [Roche: median 33 (12; 75) U/ml; Euroimmun: Median 62 (27; 100) RU/ml], followed by significantly higher IgG titres in all participants 21 days after the booster (Roche: > 250 U/ml, Euroimmun: > 100 RU/ml in all controls).

These findings are in drastic contrast to the results in the Tx group: 21 days after the prime dose, 48 out of 50 patients (96%) showed no Anti-SARS-CoV-2 IgG titres above the thresholds of the three tests used; for 45 of these patients, the results did not change 3 weeks following the boosting dose (Fig. 1a–c). One patient (male heart transplant, 29 years old, 482 days post Tx, immunosuppression with tacrolimus and mycofenolate) had IgG antibody titres comparable to the control group after boost dose, the other four patients showed a weak antibody response, with titres above the cut-off values, but markedly lower than the lowest response among the control group. Results were consistent for all three tests (Abbot, Roche, Euroimmun) used.

#### Neutralizing antibodies against SARS-CoV-2 (Fig. 2)

**Fig. 2:**
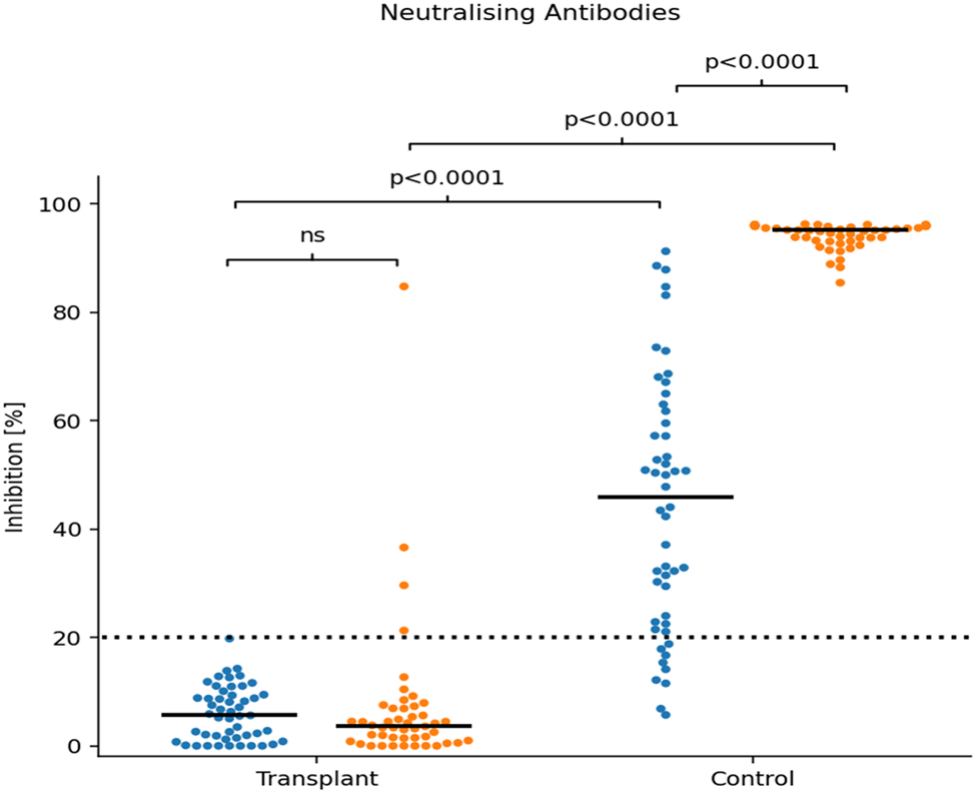
Serological responses to BNT162b2 vaccination in transplant recipients and healthy controls (*n* = 50 each): Inhibitory capacity of neutralizing antibodies. Samples drawn 21 days after the prime (blue dots) and the booster dose (orange dots). Scatter plots show inhibition in %. Statistical analysis was by Mann–Whitney Test. Horizontal solid lines show Medians. Horizontal dotted lines show cut-off values according to manufacturer (Genscript). *Ns* non-significant

The analysis of the functional inhibitory capacity of neutralizing anti-SARS-CoV-2 antibodies demonstrated a positive immunization effect (cut-off ≥ 20% inhibition) in 82% of control individuals after the prime dose (with a large scatter of response) and in all controls after the second dose [median 95% (93;96) boost vs 46% (23;62) prime, *p* < 0.0001].

In contrast, no Tx patient showed a positive inhibitory capacity after prime dose, with no significant increase after the boost dose [median 4% (1; 7) after boost, < 0.0001 vs control]. Consistent with the findings of Anti-SARS-CoV-2 IgGs only one patient showed an inhibition comparable to healthy controls.

### T-cell response following boost SARS-CoV-2 vaccination

**Fig. 3:**
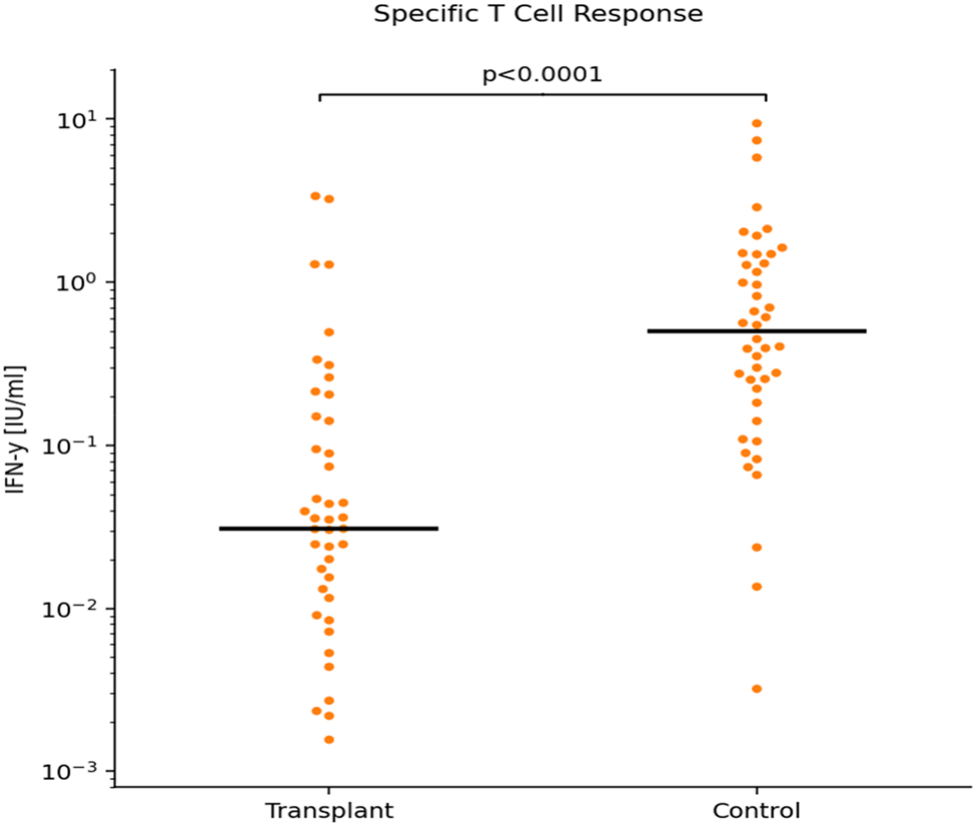
Specific T-cell response as stimulated IFN-γ release. Samples drawn 21 days after the booster dose. Scatter plots show Interferon γ (IFN-γ) release after stimulation with SARS-CoV-2 specific peptides (Miltenyi Biotec), given in International Units per ml [IU/ml]. Statistical analysis was by (Mann–Whitney Test). Horizontal lines show Medians. *ns *non-significant

The Interferon (IFN)-γ response to spike antigens SARS-CoV-2 peptides in whole blood samples drawn 21 days after the boost dose was significantly lower in the Tx patient group [median 0.031 (0.007; 0.141)] when compared to controls [median 0.512 (0.172; 1.281), *p* < 0.0001)]. However, there was an overlap between groups (Fig. 3).

Eight Tx patients with no detectable antibodies after boost dose did show an IFN-γ release of > 0.16 IU/ml (suggested as a cut-off for scoring by Petrone et al. [16]) In 80% of controls IFN-γ release was > 0.16 IU/ml.

There was no significant difference in the relatively low IFN-γ production of unstimulated whole blood samples between the groups.

## Discussion

This study demonstrates a lack of immunogenicity of the completed prime-boost vaccination with the mRNA SARS-CoV-2 vaccine BNT162b2 in cardiothoracic transplant recipients even 3 weeks after the second dose, strongly suggesting that immunosuppressed cardiothoracic organ transplant recipients are left immunologically unprotected against COVID-19 infection.

Reduced immune responses to conventional vaccination concepts following organ transplantation [13, 14] or in general, for patients under immunosuppressive therapy [17] have been reported before. However, the extent of missing humoral and cellular immune response following vaccination appears unexpected.

First insights into the immunogenicity of the BNT162b2 vaccine in an immunocompromised patient population have been reported as interim results from the SOAP-trial on cancer patients: the immune response following the prime dose was low in solid cancer patients (< 40%) and very low in haematological cancer patients (< 15%). However, in their population efficacy was greatly increased by boosting after 21 days [18].

There have been recent reports on poor anti-spike (S) antibody responses to mRNA vaccines in renal [19] and liver [20] transplant patients as well as in a mixed cohort of single organ transplant recipients [21] all of which had included patients over a wide range of years post-transplant, with reported semiquantitative serologic testing only. We present more detailed data on B-cell as well as specific T-cell responses in an uniform group of thoracic organ transplant recipients, all in their 2nd–3rd year post-transplant.

In our study, all participants have completed a full two-dose vaccination regimen, the doses being exactly 21 days apart: it demonstrates no seroconversion following the completed two-dose vaccination strategy in the vast majority (90%) of tested cardiothoracic organ recipients. These results contrast with the robust immunogenicity in the control group, who already exhibited a 98% seroconversion following the prime dose (although with a wide scatter of antibody titres), followed uniformly by a significant, on average > tenfold increase of IgG as well as neutralizing antibodies after the boosting.

In contrast to the healthy control group, evidence for a specific T-cell response (as determined by IFN-γ release of whole blood stimulated by spike antigens SARS-CoV-2 peptides) was also lacking in the majority of transplant recipients. However, in a subgroup of transplant recipients—with no detectable humoral response—a small IFN-γ release could be observed. Although cross-reactivity with a former Corona Virus-infection cannot be ruled out as a possible explanation [16], it might give evidence for a weak specific T-cell response in this subgroup of patients. The detection of specific T-cell responses in individuals lacking detectable circulation antibodies has also been described in convalescents after asymptomatic to mild COVID-19 infections [22]. The authors conclude that seroprevalence as an indicator may underestimate the extent of adaptive immune responses against SARS-CoV-2. The importance to combine analysis of B- and T-cell immunity has been emphasized elsewhere [23, 24]. In spite of growing insights into the persistence and decay of antibody responses both following infection [25] and vaccination, [9, 26] we do not yet know the exact correlates of immunity neither regarding the levels of required antibody titres nor whether suboptimal B-cell responses combined with T-cell responses might still protect from severe COVD-19.

**Limitations** of our study include the small number of patients enrolled. Larger populations are necessary to answer additional questions:

Considering the time-dependent and distinct immunosuppressive regimens after single organ transplantation, it seems obvious that doses and composition of different immunosuppressive strategies may impact on the immunogenicity after mRNA vaccination against SARS-CoV-2.

Our findings focussed on cardiothoracic patients in their first three years post-transplant, most of them being on triple immunosuppressive therapy including a calcineurin inhibitor, mycophenolate-mofetil as well as corticosteroids. The relative high-maintenance immunosuppression might explain why our finding of poor humoral response was even more pronounced than recent reports by others: in a small group of 23 renal patients, the five patients with (low) detectable antibodies were on average 18 years post-transplant [19]; in a cohort of 80 liver transplants (median of 5 years post-transplant, 47% with (low-titre) detectable antibodies) maintenance immunosuppression was lower compared to our study group, with anti-metabolite agents included in only 50% of patients, and only 21% of patients being on triple immunosuppressive therapy [20]. In a larger mixed cohort of solid organ transplant recipients poor humoral response was associated with older age, cardiothoracic transplant organ, first years post-transplant, maintenance immunosuppression regime including anti-metabolites [21]—all factors holding true for our study population.

Larger scale analyses have to elucidate whether long-term thoracic organ transplant recipients under lowered maintenance immunosuppression may confer better vaccination effects. Future studies will also have to focus on age per se. In fact, we observed mild antibody responses to BNT162b2 in younger transplant recipients.

Our sobering results on the poor response to the mRNA BNT162b2 vaccine in transplant recipients prompt further questions on dosing of the vaccine. The preliminary data by Boharsky et al. suggest that the mRNA-1273 SARS-CoV-2 vaccine by Moderna with a higher concentration per dose may confer immune responses in a larger percentage of transplants [21], but this certainly needs deeper investigation.

To gain adequate protection against other potentially threatening infections augmenting vaccination strategies such as higher doses per vial or additional boosting have been suggested for transplant recipients before [13, 27, 28]./ Considering the beneficial data on safety and adverse local and systemic events of the BNT162B2 vaccine in immunocompromised cancer [18] and transplant [29] patients, additional booster dose(s) could be considered, at least in those transplant patients showing at least some detectable B- or T-Cell-response to the first two doses. Of course, additional information on the effectiveness of other COVID-19 vaccines, e.g. vector-based vaccines, is needed.

In summary, given the globally poor antibody- and T-cell response of our transplant patients to a completed two-dose regimen with the mRNA BNT162b2 vaccine our findings mandate an urgent review of vaccination strategies for organ transplant recipients. As there may be relevant differences in immune responses among immunosuppressed patients depending on age, time since transplant, immunosuppressive regimen etc., post-vaccination testing for both, B- and T-cell responses is advisable for best medical care.

As long as transplant recipients are left unprotected, adherence to all public health measures in place, such as social distancing and shielding even after vaccination is mandatory. Creating herd immunity around these patients using a strategy of “ring vaccination” should be an additional safety measure.

## Data Availability

The data underlying this article will be shared on reasonable request to the corresponding author.

## References

[1] Zhou F, Du YuT, R, Fan G, Liu Y, Liu Z, Xiang J, Wang Y, Song B, Gu X, Guan L, Wie Y, Li H, Wu X, Xu J, Tu S, Zhang Y, Chen H, Cao B (2020). Clinical course and risk factors for mortality of adult inpatients with COVID-19 in Wuhan, China: a retrospective cohort study. Lancet.

[2] Kates OS, Haydel BM, Florman SS, Rana MM, Chaudhry ZS, Ramesh MS, Safa K, Kotton CN, Blumberg EA, Besharatian BD, Tanna SD, Ison MG, Malinis M, Azar MM, Rakita RM, Morillas JA, Majeed A, Sait AS, Spaggiari M, Hemmige V, Mehta SA, Neumann H, Badami A, Goldman JD, Lala A, Hemmersbach-Miller M, McCort ME, Bajrovic V, Ortiz-Bautista C, Friedman-Moraco R, Sehgal S, Lease ED, Fisher CE, Limaye AP (2020). COVID-19 in solid organ transplant: a multi-center cohort study. Clin Infect Dis.

[3] Caillard S, Chavarot N, Francois H, Matignon M, Greze C, Kamar N, Gatault P, Thaunat O, Legris T, Frimat L, Westeel PF, Goutaudier V, Jdidou M, Snanoudj R, Colosio C, Sicard A, Bertrand D, Mousson C, Bamoulid J, Masset C, Thierry A, Couzi L, Chemouny JM, Duveau A, Moal V, Blancho G, Grimbert P, Durrbach A, Moulin B, Anglicheau D, Ruch Y, Kaeuffer C, Benotmane I, Solis M, LeMeur Y, Hazzan M, Danion F, French SOT COVID Registry (2021). Is Covid-19 infection more severe in kidney transplant recipients?. Am J Transplant.

[4] Pereira MR, Mohan S, Cohen DJ, Husain SA, Dube GK, Ratner LE, Arcasoy S, Aversa MM, Benvenuto LJ, Dadhania DM, Kapur S, Dove LM, Brown RS, Rosenblatt RE, Samstein B, Uriel N, Farr MA, Satlin M, Small CB, Walsh TJ, Kodiyanplakkal RP, Miko BA, Aaron JG, Tsapepas DS, Emond JC, Verna EC (2020). COVID-19 in solid organ transplant recipients: initial report from the US epicentre. Am J Transplant.

[5] Latif F, Farr M, Clerkin K, Habal M, Takeda K, Naka Y, Resaino S, Sayer G, Uriel N (2020). Characteristics and outcomes of recipients of heart transplant with coronavirus disease. JAMA Cardiol.

[6] Castells MC, Philippis EJ (2021). Maintaining safety with SARS-CoV-2 vaccines. N Engl J Med.

[7] Polack F, Thomas S, Kitchin N, Absalon J, Gurtman A, Lockhart S, Perez J, Pérez Marc G, Moreira E, Zerbini C, Bailey R, Swanson K, Roychoudhury S, Koury K, Li P, Kalina W, Cooper D, Frenck R, Hammitt L, Türeci Ö, Nell H, Schaefer A, Ünal S, Tresnan D, Mather S, Dormitzer P, Şahin U, Jansen K, Gruber W (2020). Safety and efficacy of the BNT162b2 mRNA covid-19 vaccine. N Engl J Med.

[8] Baden LR, El Sahly HM, Essink B, Kotloff K, Frey S, Novak R, Diemert D, Spector SA, Rouphael N, Creech CB, McGettigan J, Khetan S, Segall N, Solis J, Brosz A, Fierro C, Schwartz H, Neuzil K, Corey L, Gilbert P, Janes H, Follmann D, Marovich M, Mascola J, Polakowski L, Ledgerwood J, Graham BS, Bennett H, Pajon R, Knightly C, Leav B, Deng W, Zhou H, Han S, Ivarsson M, Miller J, Zaks T (2021). Efficacy and safety of the mRNA-1273 SARS-CoV-2 vaccine. N Engl J Med.

[9] Walsh EE, Frenck R, Falsey AR, Kitchin N, Absalon J, Gurtman A, Lockhart S, Neuzil K, Mulligan MJ, Bailey R, Swanson KA, Li P, Koury K, Kalina W, Cooper D, Fontes-Garfias C, Shi PY, Türeci Ö, Thompkins KR, Lyke KE, Raabe V, Dormitzer PR, Jansen KU, Sahin U, Gruber WC (2020). Safety and immunogenicity of two RNA-based COVID-19 vaccine candidates. N Engl J Med.

[10] Aslam S, Goldstein DR, Vos R, Gelman AE, Kittleson MM, Wolfe C, Danziger-Isakov L (2021). Covid-19 vaccination in our transplant recipients: the time is now. J Heart Lung Transplant.

[11] Guidance from the International Society of Heart and Lung Transplantation regarding the SARS CoV-2 pandemic: SARS-CoV-2 vaccination in heart and lung transplantation, recommendations from the ISHLT COVID-19 task force, revised March 15, 2021. https://community.ishlt.org.

[12] Danziger-Isakov L, Kumar D (2019). Vaccination of solid organ transplant candidates and recipients: guidelines from the American society of transplantation infectious diseases community of practice. Clin Transplant.

[13] Haddadin Z, Krueger K, Thomas LD, Overton ET, Ison M, Halasa N (2021). Alternative strategies of posttransplant influenza vaccination in adult solid organ transplant recipients. Am J Transplant.

[14] Admon D, Engelhard D, Strauss N, Goldman N, Zakay-Rones (1997). Antibody response to influenza immunization in patients after heart transplantation. Vaccine.

[15] Fischer B, Lindenkamp C, Lichtenberg C, Birschmann I, Knabbe C, Hendig D (2021). Evidence of long-lasting humoral and cellular immunity against SARS-CoV-2 even in elderly COVID-19 convalescents showing a mild to moderate disease progression. medRxiv.

[16] Petrone L, Petruccioli E, Vanini V, Cuzzi G, Najafi Fard S, Alonzi T, Castilletti C, Palmieri F, Gualano G, Vittozzi P, Nicastri E, Lepore L, Antinori A, Vergori A, Caccamo N, Cantini F, Girardi E, Ippolito G, Grifoni A, Goletti D (2021). A whole blood test to measure SARS-CoV-2-specific response in COVID-19 patients. Clin Microbiol Infect.

[17] Rondaan C, Furer V, Heijstek MW, Agmon-Levin N, Bijl M, Breedveld FC, D'Amelio R, Dougados M, Kapetanovic MC, van Laar JM, Ladefoged de Thurah A, Landewé R, Molto A, Müller-Ladner U, Schreiber K, Smolar L, Walker J, Warnatz K, Wulffraat NM, van Assen S, Elkayam O (2019). Efficacy, immunogenicity and safety of vaccination in adult patients with autoimmune inflammatory rheumatic diseases: a systematic literature review for the 2019 update of EULAR recommendations. RMD Open.

[18] Monin-Aldama L, Laing AG, Munoz-Ruiz M, McKenzie DR, del Barrio M, Alaguthurai T, Domingo-Vila C, Hayday TS, Graham C, Seow J, Abdul-Jawad S, Kamdar S, Harvey-Jones E, Graham R, Cooper J, Khan M, Vidler J, Kakkassery H, Shubhankar S, Davis R, Dupont L, Quijorna IF, Lee P, Eum J, Poole MC, Joseph M, Davies D, Wu Y, Montes A, Harries M, Rigg A, Spicer J, Malim MH, Fields P, Patten P, Di Rosa F, Papa S, Tree T, Doores K, Hayday AC, Irshad S (2021). Interim results of the safety and immune-efficacy of 1 versus 2 doses of COVID-19 vaccine BNT162b2 for cancer patients in the context of the UK vaccine priority guidelines. medRxiv.

[19] Korth J, Jahn M, Dorsch O, Anastasiou OE, Sorge-Hädicke B, Eisenberger U, Gäckler A, Dittmer U, Witzke O, Wilde B, Dolff S, Kribben A (2021). Impaired humoral response in renal transplant recipients to SARS-CoV-2 vaccination with BNT162b2 (Pfizer-BioNTech). Viruses.

[20] Rabinowich L, Grupper A, Baruch R, Ben-Yehoyada M, Halperin T, Turner D, Katchman E, Levi S, Houri I, Lubezky N, Shibolet O, Katchman H (2021). Low immunogenicity to SARS-CoV-2 vaccination among liver transplant recipients. J Hepatol.

[21] Boyarsky BJ, Werbel WA, Avery RK, Tobian AAR, Massie AB, Segev DL, Garonzik-Wang JM (2021). Antibody response to 2-dose SARS-CoV-2 mRNA vaccine series in solid organ transplant recipients. JAMA.

[22] Sekine T, Perez-Potti A, Rivera-Ballesteros O, Strålin K, Gorin JB, Olsson A, Llewellyn-Lacey S, Kamal H, Bogdanovic G, Muschiol S, Wullimann DJ, Kammann T, Emgård J, Parrot T, Folkesson E, Rooyackers O, Eriksson LI, Henter JI, Sönnerborg A, Allander T, Albert J, Nielsen M, Klingström J, Gredmark-Russ S, Björkström NK, Sandberg JK, Price DA, Ljunggren HG, Aleman S, Buggert M (2021). Robust T cell immunity in convalescent individuals with asymptomatic or mild COVID-19. Cell.

[23] Cox RJ, Brokstad KA (2020). Not just antibodies: B cells and T cells mediate immunity to COVID-19. Nat Rev Immunol.

[24] Sauer K, Harris T (2020). An effective COVID-19 vaccine needs to engage T cells. Front Immunol.

[25] Iyer AS, Jones FK, Nodoushani A, Kelly M, Becker M, Slater D, Mills R, Teng E, Kamruzzaman M, Garcia-Beltran WF, Astudillo M, Yang D, Miller TE, Oliver E, Fischinger S, Atyeo C, Iafrate AJ, Calderwood SB, Lauer SA, Yu J, Li Z, Feldman J, Hauser BM, Caradonna TM, Branda JA, Turbett SE, LaRocque RC, Mellon G, Barouch DH, Schmidt AG, Azman AS, Alter G, Ryan ET, Harris JB, Charles RC (2020). Persistence and decay of human antibody responses to the receptor binding domain of SARS-CoV-2 spike protein in COVID-19 patients. Sci Immunol.

[26] Widge AT, Rouphael NG, Jackson LA, Anderson EJ, Roberts PC, Makhene M, Chappell JD, Denison MR, Stevens LJ, Pruijssers AJ, McDermott AB, Flach B, Lin BC, Doria-Rose NA, O'Dell S, Schmidt SD, Neuzil KM, Bennett H, Leav B, Makowski M, Albert J, Cross K, Edara VV, Floyd K, Suthar MS, Buchanan W, Luke CJ, Ledgerwood JE, Mascola JR, Graham BS, Beigel JH (2021). mRNA-1273 Study Group. Durability of responses after SARS-CoV-2 mRNA-1273 vaccination. N Engl J Med.

[27] Natori Y, Shiotsuka M, Slomovic J, Hoschler K, Ferreira V, Ashton P, Rotstein C, Lilly L, Schiff J, Singer L, Humar A, Kumar D (2018). A Double-blind, randomized trial of high-dose vs standard-dose influenza vaccine in adult solid-organ transplant recipients. Clin Infect Dis.

[28] Cordero E, Roca-Oporto C, Bulnes-Ramos A, Aydillo T, Gavaldà J, Moreno A, Torre-Cisneros J, Montejo JM, Fortun J, Muñoz P, Sabé N, Fariñas MC, Blanes-Julia M, López-Medrano F, Suárez-Benjumea A, Martinez-Atienza J, Rosso-Fernández C, Pérez-Romero P (2017). Two doses of inactivated influenza vaccine improve immune response in solid organ transplant recipients: results of TRANSGRIPE 1–2, a randomized controlled clinical trial. Clin Infect Dis.

[29] Ou MT, Boyarsky BJ, Motter JD, Greenberg RS, Teles AT, Ruddy JA, Krach MR, Jain VS, Werbel WA, Avery RK, Massie AB, Segev DL, Garonzik-Wang JM (2021). Safety and reactogenicity of 2 doses of SARS-CoV-2 vaccination in solid organ transplant recipients. Transplantation.

